# Reinventing Diagnostics for Personalized Therapy in Oncology

**DOI:** 10.3390/cancers2021066

**Published:** 2010-06-02

**Authors:** Diponkar Banerjee

**Affiliations:** 1 Centre for Translational and Applied Genomics (CTAG), Provincial Health Services Authority (PHSA) Laboratories, Vancouver, British Columbia, Canada; 2 Department of Pathology and Laboratory Medicine, Faculty of Medicine, University of British Columbia, Vancouver, British Columbia, Canada; 3 Department of Pathology, British Columbia Cancer Agency (BCCA), 600 West 10th Avenue, Vancouver, British Columbia, V5Z4E6, Canada; E-Mail: dbanerje@bccancer.bc.ca; Tel.: +1-604-877-6074; Fax: +1-604-877-6017

**Keywords:** lung, breast, genomics, classification, biomarkers, personalized therapy

## Abstract

Human cancers are still diagnosed and classified using the light microscope. The criteria are based upon morphologic observations by pathologists and tend to be subject to interobserver variation. In preoperative biopsies of non-small cell lung cancers, the diagnostic concordance, even amongst experienced pulmonary pathologists, is no better than a coin-toss. Only 25% of cancer patients, on average, benefit from therapy as most therapies do not account for individual factors that influence response or outcome. Unsuccessful first line therapy costs Canada CAN$1.2 billion for the top 14 cancer types, and this extrapolates to $90 billion globally. The availability of accurate drug selection for personalized therapy could better allocate these precious resources to the right therapies. This wasteful situation is beginning to change with the completion of the human genome sequencing project and with the increasing availability of targeted therapies. Both factors are giving rise to attempts to correlate tumor characteristics and response to specific adjuvant and neoadjuvant therapies. Static cancer classification and grading systems need to be replaced by functional classification systems that not only account for intra- and inter- tumor heterogeneity, but which also allow for the selection of the correct chemotherapeutic compounds for the individual patient. In this review, the examples of lung and breast cancer are used to illustrate the issues to be addressed in the coming years, as well as the emerging technologies that have great promise in enabling personalized therapy.

## 1. Introduction

The diagnosis and classification of human cancers by pathologists remains largely based upon microscopic examination of tissue (histopathology) or cells (cytopathology) using brightfield microscopy of chemical dye-stained tissue sections or cytologic preparations. The compound light microscope was invented by Hans Lippershey, Zacharias Janssen, and Hans Janssen in 1590 [1]. Tissue and cellular stains were developed in the mid 1800s. The stains were largely based upon haematoxylin, a naturally occurring chemical derived from the logwood tree, *Haematoxylon campechianum.* The logwood tree was "discovered" by the Spanish in Campeche in the Yucatan peninsula in 1502, where the locals were already using extracts of the logwood tree for dyeing cotton [2]. To this day pathologists everywhere in the world rely upon haematoxylin and eosin (H&E) labelled tissue sections to diagnose cancer using a compound light microscope. Diagnostic criteria and current classification systems in clinical use for human cancers remain largely morphology based [3,4,5,6,7,8,9,10,11,12,13,14,15,16,17,18,19,20,21,22,23,24,25,26], with their attendant problems with interobserver variability [27,28,29,30,31,32,33,34,35,36,37,38,39,40,41,42,43,44,45]. Notable exceptions are the classification systems of neoplasms of haematopoietic and lymphopoietic systems, which are now heavily reliant upon ancillary technologies [46]. It would not be too far-fetched to state that, with a few exceptions, we continue to use technology that is between 400 and 500 years old [47].

Since the early days of histology, in a gradual and incremental fashion, additional staining methods were developed to better define tissue components and cellular detail. In the 1960s, the arrival of diagnostic transmission electron microscopy [48] changed some aspects of cancer pathology, only to be largely supplanted by immunohistochemistry and molecular diagnostics. The latter two were due to the invention of monoclonal antibody technology by Kohler and Milstein in 1975 [49], and the polymerase chain reaction by Kary Mullis in 1986 [50], respectively. These two inventions accelerated the rate of change in how ancillary methods were introduced in clinical laboratories, a chain reaction in itself. The availability of monoclonal antibodies against lineage-specific antigens expressed in haematopoietic cells and the invention, in the 1960's, of fluorescence activated cell sorting and flow cytometry by Herzenberg [51] changed the way we diagnose leukemias and lymphomas. However, morphology still plays a major role in diagnosis of these diseases. The successful completion of the human genome sequencing project [52] has now spawned many approaches to try and understand what is awry in human cancers. 

## 2. The Financial Imperative for Personalized Therapy

Canada has the second highest public expenditure on pharmaceuticals per capita amongst OECD countries (Table 1). Drug spending in this country is estimated to have reached CAN$29.8 billion in 2008, representing 17.4% of the total health expenditure [53]. Spending on pharmaceuticals has more than doubled over the past 10 years. Its growth is faster than that of healthcare costs generated by hospitals, physicians, and other health professionals. Unfortunately, matching the right therapy to the individual patient is not easy and tends to be a trial and error approach. The outcome of individual patients is unpredictable because the probabilities are based upon averages. The possibility of over- or under- treatment exists, with consequences including unnecessary toxicity or denial of more efficacious therapies. It is estimated that between 25% and 80% of drugs used to treat various chronic diseases have clinical efficacy, with oncology dugs being in the lowest efficacy group at 25% [54]. I have estimated the direct costs of treating the top 14 cancers in Canada and ranked these by the cost of treating cancers that fail to respond, based upon crude five year mortality rates (Table 2). These costs include standard courses of radiation therapy and chemotherapy, but not surgical costs, costs of second or third line therapy, or indirect costs associated with morbidity and hospitalization. While this may not be sophisticated health economics analysis, it provides a snap shot of the costs of ineffective therapies in Canada every year, supported by the tax-paying citizens of this country. This represents CAN$1.2 billion dollars being spent on therapies that do not work on individual patients. Imagine a new paradigm that allows personalized therapy, which could redirect CAN$1.2 billion of wasted resources towards matching the right therapy to the right patient without adding to the annual expenditures of a cash-strapped publicly funded healthcare system. Extrapolating this to the global cancer burden (10.9 million new cases per year in 2002) [55] would mean wasted resources of CAN$90 billion per year if all countries followed current standard therapies for the top 14 cancers.

In this review, I summarize the most recent literature on understanding human cancers by using genomic tools. I describe how genomics information is beginning to change our thinking about classification systems, and prognostic and predictive factors. I make a case for the need to incorporate genomics technology into the clinical laboratory. I discuss the limitations of fixed morphologic classification systems and argue for the development of functional classification systems that account for the individual variation and plasticity of cancer stem cells by using lung and breast cancer cells as examples. 

**Table 1:** Ranking of OECD countries by spending (in US dollars) on pharmaceuticals per capita (from OECD Health Data 2009, November 2009) (http://www.oecd.org/document/16/0,3343,en_2649_34631_2085200_1_1_1_1,00.html).

Rank	OECD Country	Public $ per capita	Total $ per capita	Percentage public
1	United States	307	1,015	30.2
**2**	**Canada**	**302**	**770**	**39.2**
3	Belgium	353	703	50.1
4	France	472	679	69.4
5	Spain	464	642	72.3
6	Japan	436	609	71.7
7	Germany	447	602	74.3

**Table 2:** Cancer Incidence in Canada in 2009, Ranked by Cost of Treatment Failure (in Canadian dollars) per year.

	Cancer	Total	Per case cost	Annual Canadian Costs ^a^	Crude 5 year Survival rates (%)	Mortality at 5 years (%)	Cost of treatment failure
1	Lung	23,400	$27,295	$638,703,000	14	86	$549,284,580
2	Colorectal	22,000	$26,742	$588,324,000	66	34	$200,030,160
3	Pancreas	3,900	$29,395	$114,640,500	7	93	$106,615,665
4	Lymphoma	7,200	$23,759	$171,064,800	57	43	$73,557,864
5	Ovary	2,500	$40,666	$101,665,000	41	59	$59,982,350
6	Leukemia	4,700	$19,891	$93,487,700	57	43	$40,199,711
7	Kidney	4,600	$27,958	$128,606,800	60	40	$51,442,720
8	Head/Neck*	9,250	$19,891	$183,991,750	74	26	$47,837,855
9	Breast	22,900	$12,156	$278,372,400	89	11	$30,620,964
10	Bladder	6,900	$13,592	$93,784,800	80	20	$18,756,960
11	Prostate	25,500	$12,156	$309,978,000	96	4	$12,399,120
12	Endometrial	4,400	$17,902	$78,768,800	87	13	$10,239,944
13	Cervix	1,300	$22,212	$28,875,600	78	22	$6,352,632
14	Melanoma	5,000	$5,304	$26,520,000	94	6	$1,591,200
	Total Cost of treatment failure						$1,208,911,725
	Top 5 cancers						$989,470,619
	Top 10 cancers						$1,178,328,829

^a^ Based on extrapolation from US National Cancer treatment costs, converted to CAN$; * Includes oral, thyroid and larynx.

## 3. Lung Cancer

Lung cancer is the most common cause of cancer-related mortality affecting men and women. The World Health Organization estimated that in 2004, 1.3 million deaths occurred globally due to lung cancer [56]. In the United States of America, an estimated 219,440 new cases were diagnosed, and 159,390 deaths due to lung cancer were expected in 2009 [57]. 

Generally, pathologists have attempted to use morphology and immunohistochemistry to broadly categorize lung cancer into small cell carcinoma (SCLC), and non-small cell lung cancer (NSCLC). The latter includes adenocarcinoma (ADC), squamous cell carcinoma (SCC), and large cell carcinoma (LCC) subtypes [58]. The LCC category has been further subdivided into sarcomatoid, lymphoepithelial, clear-cell, rhabdoid, basaloid, and large cell neuroendocrine carcinoma subtypes [59]. Unfortunately, even experienced pathologists with an interest in lung cancer cannot agree on the identification of NSCLC subtypes in preoperative samples, with an accuracy of 55% or less, depending upon the publication [60,61]. This is essentially no better than a coin-toss. Histology does not predict outcomes in NSCLC treated with combined vinorelbine or gemcitabine and cisplatin regimens [62]. This is not surprising, given the lack of agreement amongst expert pathologists. However, other publications and meta-analysis of previously published studies suggest that histological subtyping of NSCLC can predict responses to modern chemotherapeutic agents [58,63]. This has led to attempts to better define subsets of NSCLC by using immunohistochemically defined biomarkers, gene expression signatures, gene copy number variation, mutation analysis of growth factor receptor genes, and microRNA profiling.

### 3.1. Immunohistochemistry in NSCLC

Subtyping of NSCLC using immunohistochemistry involves the use of antibodies directed against thyroid transcription factor-1 (TTF-1), p63, CK7, and high molecular weight cytokeratins (HMWCK) [58]. The problem is that no single marker reliably excludes or includes a subtype as a possibility. A combination matrix of biomarkers increases the probability of correctly classifying a given tumor. A recurrent problem with attempts to define biomarkers for cancer classification is that biomarkers are invariably judged against standard morphologic criteria, which as I have already discussed, are inadequate. Although TTF-1 is expressed predominantly in lung adenocarcinomas, it is also expressed in 5% to 21% of squamous cell carcinomas [64,65]. P63 is expressed in 97% of SCC, 30% of adenocarcinomas, 50% of large cell neuroendocrine carcinomas, and 77% of small cell carcinomas [66].

Another protein, desmocollin-3, a desmosome associated protein, is expressed in about 50% of undifferentiated large-cell lung cancers, 100% of basaloid carcinomas, and almost 60% of clear-cell carcinomas, but is not expressed in sarcomatoid carcinomas [67].

### 3.2. Gene Expression Profiling in NSCLC

Molecular profiling was able to correctly classify a small group of patients with NSCLC on the basis of relapse *vs.* relapse-free survival [68]. However, these genes were not significant in an RT-PCR validation study [69]. A 31 gene signature is able to correctly predict lymph node metastasis in 85% of NSCLC patients [70]. A "metagene" model has been developed to predict recurrence of stage IA NSCLC. Univariate and multivariate analyses showed that the model predicted recurrence significantly better than stage, tumor diameter, nodal status, age, sex, histologic subtype, or smoking history, with an overall predictive accuracy of 79 percent [71]. 

Mitotic kinesin KIF14 is an independent prognostic factor for disease-free survival, including stage, differentiation, and histology in multivariate analysis [72]. Another 35-gene signature stratified patients with NSCLC at stage 1A into distinct prognostic subgroups, and overexpression of the encoded proteins of 2 of the genes, *TAL2* (T-cell acute lymphocytic leukemia 2) and *ILF3* (interleukin enhancer binding factor 3), was detected in the tumors [73].

### 3.3. SAGE Transcriptome Profiles in Carcinoma-in-Situ and Invasive NSCLC

Lonergan and others reported the first large scale transcriptomic profiling of carcinoma-in-situ (CIS) of the lung, invasive squamous cell carcinoma (SCC), and precancerous (PC) metaplastic and dysplastic epithelia. They identified genes associated with epidermal development and xenobiotic metabolism/detoxification in CIS lesions, genes associated with the immune response, and genes linked with tissue remodeling/fibrosis in SCC. In addition, they observed down-regulation of genes associated with mucociliary differentiation in CIS and PC lesions [74].

### 3.4. Gene Copy Number Variation

A number of genes with copy number increases or overexpression of their encoded proteins, or both, are prognostically significant in NSCLC. *Skp2* copy number increase is seen predominantly in SCC, with either gains or losses in ADC. Skp2 protein overexpression, but not *skp2* copy number, accompanied by *ras* mutations, is associated with poor prognosis [75]. 

Telomerase gene *hTERT* mRNA overexpression is more frequent in SCC than in ADC, and is associated with *hTERT* amplification in ADC. *HTERT* amplification is an independent prognostic marker for shorter recurrence-free survival in ADC [76]. 

Although *MET* amplification is relatively uncommon, it is detected more often in SCC than in ADC, and predicts worse survival in SCC in multivariate analysis [77]. 

A controversial area is the correlation between *EGFR* copy number in NSCLC and response to tyrosine kinase inhibitors (TKIs), such as erlotinib and gefitinib. Patients with *EGFR* copy number gains have higher overall response rates than those without gains in placebo-controlled trials of TKIs. *EGFR* copy number has been claimed to be a stronger predictor of response than *EGFR* mutation status [78,79]. On the other hand, a meta-analysis of over 200 published studies showed that *EGFR* mutations are more predictive of response to single-agent epidermal growth factor receptor TKIs in advanced NSCLC than *EGFR* copy number gains. This correlation was seen predominantly in whites and less so amongst Asians [80].

### 3.5. Mutation of Tyrosine Kinase Domains in the Epidermal Growth Factor Receptor (EGFR) Gene

Acquired activating mutations in the tyrosine kinase domain encoding region of EGFR have been identified in lung cancer. It is claimed that these mutations can be used to predict responsiveness to tyrosine kinase inhibitors (TKIs), such as gefitinib and erlotinib [81,82,83]. Such mutations are more frequently associated with adenocarcinoma (especially of the bronchioloalveolar type), females, Asians, and patients who have never smoked [81]. However, these early studies had no control group. Therefore, there was concern whether the association of activating mutations within the *EGFR* TK domain with histology, gender, ethnic origin, and “never smoked” status was responsible for the response to TKIs, rather than for the mutation status itself. In fact, when tumors from patients in a clinical trial of erlotinib *versus* placebo were tested for *EGFR* mutations, expression, and gene copy number, multivariate analysis showed that the expression of EGFR protein, but not mutation status or copy number, was associated with response. Survival after treatment was not predicted by the status of EGFR expression, the *EGFR* gene copy number, or mutation status [78]. The story gets more complicated as other studies showed that 10% of cases without activating EGFR mutations respond to TKIs [84]. TKI therapy, however, may be detrimental to unselected patients not stratified by mutational analysis [85].

### 3.6. MicroRNAs in Typing of NSCLC

MicroRNAs (miRNA) are short non-coding RNA genes that regulate gene expression by either translational down-regulation or by degradation of target mRNA [86]. When miRNAs hsa-miR-205, hsa-miR-21, and U6 snRNA were measured by quantitative reverse transcription-PCR in SCC and NSCLC, a formula based upon average cycle thresholds (C_T_) could accurately distinguish between SCC and NSCLC, even in pre-resection biopsies [87]. 

Human serum contains circulating miRNAs. Genome-wide serum miRNA screening was used to determine a serum miRNA signature that correlates with survival. The levels of miR-486, miR-30d, miR-1, and miR-499 were found to be significantly associated with overall survival [88]. The combined detection of microRNAs miR-21, miR-486, miR-375, and miR-200b was able to correctly distinguish between sputum samples from lung adenocarcinoma patients and normal subjects with 80.6% sensitivity and 91.7% specificity [89]. It is likely that miRNAs will be widely used for both diagnosis and prognosis in the future.

## 4. Breast Cancer

### 4.1. Gene Expression Profiling

The current methods of assessing breast cancer tissues are largely morphology based and only a small set of biomarkers consisting of estrogen receptors (ER), progesterone receptors (PR), Her2neu protein or HER2neu gene amplification, and to limited extent, Ki-67, a marker of cell proliferation, are in routine clinical use. 

The early excitement around transcriptome profiling or gene expression profiling of breast cancer [90,91,92,93,94,95,96,97,98,99,100,101,102] has dissipated somewhat, due to concerns that predictive gene signatures may be only slightly more useful than morphology, hormone receptor status, Her2neu status, and proliferation rates. The field is still controversial, as reproducibility of gene expression data has been less than satisfactory. This is true even for the reanalysis of published data sets, let alone the data from the same analytic platforms to study different sets of tumors from different groups of patients by different groups of investigators [103,104,105,106,107].

There are a number of lessons learned from earlier gene expression studies. One is that each patient's tumor profile is somewhat unique, such that profiles for two different tumor samples from the same patient are more alike than either is to any other patient's breast cancer sample [90]. The second is that this individual "molecular portrait" is remarkably stable over time, even after exposure to chemotherapy [90]. 

Despite the observation of individual heterogeneity in gene signature patterns, it is possible to group the gene expression profiles into biological clusters. The largest is the proliferation cluster which correlates with the mitotic rate, an important parameter used in the Nottingham score grading system [108] for breast cancer. This cluster includes the genes encoding Ki-67 and PCNA, which are commonly used immunohistochemical markers for proliferating cells. The other clusters, with good correlation with immunohistochemistry (IHC) biomarker test results, include estrogen receptor pathway gene expression levels, and Her2neu. Five subtypes were defined using hierarchical clustering—luminal subtypes A and B, normal breast-like, ERBB2+, and basal-like [92]. 

Immunohistochemistry, using a simple panel of antibodies, can reproduce the molecular subtyping of breast cancers [109,110]. Torsten Nielsen and others have demonstrated that the luminal A and B subtypes are characterized by ER positivity [110]. The ERBB2+ type characterized by Her2neu protein overexpression or Her2neu gene amplification can be easily recognized using IHC and fluorescence *in situ* hybridization (FISH) methods, respectively. The basal-like type can also be identified by immunohistochemistry, as this subtype is negative for estrogen receptor and HER2, but positive for basal cytokeratins, HER1, and/or c-KIT. They studied 930 patients with 17 years mean follow-up, and found that basal cytokeratin expression was associated with low disease-specific survival. HER1 expression was observed in 54% of cases positive for basal cytokeratins (*versus* 11% of negative cases). Such cases were associated with poor survival, independent of nodal status and tumor size. C-KIT expression was more common in basal-like tumors than in other types of breast cancers, but had no prognostic value [110]. They later reported that in basal-like breast cancers, a small heat shock protein, alpha-basic-crystallin (alphaB-crystallin), was commonly expressed and associated, independent of other prognostic markers [111], with poor survival in breast cancer patients. The same group has been able to distinguish between luminal A and B subtypes by IHC, and has compared those classified by gene expression profiling and IHC by using ER, PR, Ki-67, and Her2neu labelling. The Ki67 index cut point to distinguish luminal B from luminal A tumors was 13.25%. Luminal B and luminal-HER2-positive breast cancers were associated with poor breast cancer recurrence-free and disease-specific survival in every adjuvant systemic treatment category. Of great interest was the observation that for women who received tamoxifen as their only adjuvant systemic therapy, the 10-year breast cancer-specific survival was 79% for luminal A, 64% for luminal B, and 57% for luminal-HER2 subtypes [112]. Seven molecular subtypes of breast cancer have been described with different clinical behaviors [113]. 

### 4.2. Gene Expression Profiling and Response to Neoadjuvant Therapies

In the neoadjuvant setting, predicting response by gene expression profiling has been controversial. In an early study, 10 patients (20 samples) who were to receive neoadjuvant chemotherapy had fine needle aspiration (FNA) biopsies done for gene expression profiling. Three pre-treatment FNA samples out of the 20 yielded an insufficient percentage of tumor cells for analysis. Thirty-seven genes distinguishing between good and poor responders were identified, including genes involved in cell death and chemosensitivity [95]. 

Gene expression patterns define the phenotypes of inflammatory breast cancer as well as those associated with tumor hypoxia, and gene signatures can predict residual malignancy in axillary lymph nodes after neoadjuvant chemotherapy [114]. 

In another study in the setting of neoadjuvant therapy, gene expression profiles did not predict response in locally advanced breast cancer [115]. A very recent study showed predictive signatures could be obtained from FNA biopsies, but these lost significance on multivariate analysis and did not correlate with in-vitro drug sensitivity-gene expression predictors based upon NCI-60 cell lines [116]. However, another recent study showed immune signalling molecules, such as DEFA and MAP2, a microtubule-associated protein, correlate with response to neoadjuvant taxane-based therapy [117].

In a retrospective study of 300 women, Osako *et al*. [118] found 30 (10%) achieved pathological complete remission (pCR) and 22 (7%) showed progressive disease (PD) after neoadjuvant chemotherapy (anthracycline-based, taxane, or both). Multivariate analysis demonstrated that anthracycline plus taxane chemotherapy, nuclear grade 3, estrogen (ER) or progesterone receptor (PR) negativity (note that they used a 10% cut off point for positivity), and HER2-positivity were significant predictors of pCR, whereas clinical stage T3-4 and nuclear grade 3 were significant predictors of PD. They concluded that high-grade breast cancers include subsets both highly sensitive and highly resistant to cytotoxic neoadjuvant chemotherapy. ER/PR-negativity and HER2-positivity are predictive of chemosensitivity. Advanced primary tumor stage and high nuclear grade, but not ER or PR status, are predictive of chemoresistance [118].

### 4.3. Protein Expression and Subcellular Location in Breast Cancer Cells

Protein expression and subcellular location can reveal functional changes in specific proteins. Fu *et al.* used a dissociable antibody microarray (DAMA) to visualize subcellular locations of 325 proteins in seven breast cancer cell lines, and were able to demonstrate spatial distribution differences in cyclin B1 of the cancer cell lines in comparison to normal cells [119]. They also found that not all proteins occupy subcellular locations, as predicted by protein databases such as LOCATE (http://locate.imb.uq.edu.au/), a mammalian protein subcellular localization database, and the Human Protein Atlas (HPA) (http://www.proteinatlas.org/index.php). The antibodies for the arrays were obtained from Hypromatrix, Inc., which lists a repertoire of 400 antibodies that can be individually purchased, or bought as arrays for various high throughput protein screens, including phosphoprotein detection and signalling protein assessment (http://www.hypromatrix.com/).

Triple-negative breast cancers (TNBC), which are defined by a lack of expression of estrogen, progesterone, and HER2/neu receptors, comprise 15% of all breast cancers. However, they are considered to be a heterogeneous group [120,121,122]. This subtype has an aggressive behavior, poor prognosis, and is resistant to endocrine therapies [123]. 

Two groups have looked at protein profiles in TNBC using 2D DIGE (two-dimensional difference gel electrophoresis) and matrix-assisted laser desorption/ionization time-of-flight mass spectrometry (MALDI-TOFMS) [124] or reverse phase protein arrays for specific cyclin proteins, respectively [125]. 2D DIGE and MALDI-TOFMS revealed differential expression of glycolytic enzymes, such as MDH2, PGK1, TKT, Aldolase1, cytokeratins CK7, 8, 9, 14, 17, and 19, other structural proteins such as vimentin, fibronectin, and L-plastin, as well as lactoferrin, and members of the Annexin family [124]. Reverse phase protein arrays detected Cyclins B1, D1, and E1 with distinct expressions in different breast cancer subtypes. Cyclin E1 overexpression was unique to TNBC and basal-like cancers. *CCNE1* copy number was increased in basal-like breast cancers when compared to that of other types of breast cancer, whereas *CCNB1* gene copy number change was not detected in breast cancer [125].

### 4.4. Gene Copy Number and Response to Neoadjuvant Chemotherapy

Post-neoadjuvant therapy gene copy number assessment in a small number of cases (45) has been claimed to have predictive value [126]. In this study, a 158 gene set was able to predict relapse, while a 51 gene set could predict outcome in poor responders, and a 32 gene set could predict outcome in good responders [126]. 

In a comprehensive study of seven breast cancer cell lines using the submegabase-resolution tiling (SMRT) array comparative genomic hybridization (aCGH) platform with a resolution of 80 kilobases, Shadeo and Lam have shown that 75 high-level gains and 48 losses were observed. Complex alterations with several levels of change were found on chromosome arms 1p, 8q, 9p, 11q, 15q, 17q, and 20q. Approximately 60 loci containing genes associated with the epidermal growth factor family (epidermal growth factor receptor, HER2, HER3, and HER4) showed copy number changes in multiple genes in these pathways in all seven cell lines [127]. These require validation in clinical samples and correlation with response to neoadjuvant therapies.

### 4.5. Detection of Chromosomal Aneuploidies and Gene Copy Number Changes in Fine Needle Aspirates Is Diagnostic of Breast Cancer

Certain probe panels alone have been reported to be able to distinguish breast cancer from benign lesions in cells obtained by fine needle aspirates in 100% of DNA aneuploid tumors and in 66% of DNA diploid tumors, independent of all of the other parameters evaluated [128]. 

### 4.6. Translocations

Colin Collins and others analyzed brain, breast, ovary, and prostate tumors, and breast cancer cell lines by using end sequencing profiling (ESP). They showed that these cells contain a large number of sequence-ready tumor genome breakpoints. Some rearrangements may be recurrent. Sequencing and fluorescence *in situ* hybridization confirmed the translocations, co-amplifications, and complexes of multiple genomic loci, with associated molecular heterogeneity [129]. By using multi-banding FISH (mFISH), Letessier *et al.* have demonstrated 136 break-regions in breast cancer cell lines [130]. 

### 4.7. TP53 Mutations

*TP53* mutations have been correlated with p53 protein levels and chromosome 17 abnormalities (CEP 17 polysomies) in breast cancer. For instance, cells with p53 mutations showed abnormal p53 protein expression and a higher number of chromosome 17 copies than did cells without *TP53* mutations [131]. This indicates that a combination of abnormal p53 expression and cep17 polysomy can be used as a surrogate marker for *TP53* mutation. *TP53* mutations are associated with worse outcomes [132,133,134,135,136]. Direct FISH based detection of point mutations in breast cancer cells is theoretically possible, as this has been done in microbes to predict antibiotic sensitivity [137].

### 4.8. miRNA

Altered miRNA signatures in primary breast cancers and their metastasis have been observed, including the loss of tumor suppressor miRNAs (miR-206, miR-17-5p, miR-125a, miR-125b, miR-200, let-7, miR-34 and miR-31) and the overexpression of oncogenic miRNAs (miR-21, miR-155, miR-10b, miR-373 and miR-520c) [138]. VEGF expression in breast cancer cells is triggered by HIF-1 and STAT3 under the influence of miR-20b [139]. Tumor-specific miRNAs can be detected in peripheral blood samples from breast cancer patients, and blood levels of miR-195 and let-7a decreased to control levels after surgical excision of breast tumors. Specific circulating miRNAs correlate with nodal status and estrogen receptor status [140]. 

Although the detection of miRNAs in formalin fixed paraffin-embedded tissue by FISH is difficult because of their small size, Sempere *et al*. were able to detect miRNA expression in formalin-fixed paraffin sections of breast cancer tissue by using locked nucleic acid (LNA) FITC labelled DNA probes and tyramide signal amplification following binding with horseradish peroxidase conjugated to anti-FITC antibodies [141]. They found that the expression of miR-145 and miR-205 was localized to the myoepithelial/basal cell compartment of normal mammary ducts and lobules, with a reduction or complete loss of the two miRNAs in cancer cells. They also found, compared to normal cells, loss of let-7 (lethal-7) gene expression by FISH in cancer cells in both in-situ cancers (CIS) and invasive cancers. MiR-21 expression is increased in cancer cells and tumor-associated fibroblasts. This would suggest that the lack of mir-145, miR-205, let-7, and an increased expression of miR-21 could be used to distinguish between neoplastic and non-neoplastic epithelial cells in fine-needle aspiration biopsy samples.

### 4.9. Fusion Genes

Fusion events are poorly characterized in clinical breast cancer tissue. Cell line data suggest that fusion events may be quite common. Examples include *UBR4–GBL1*, *ARHGEF2–SULF2*, *AHCYL1–RAD51C*, *RAD51C–ATXN7*, *BCAS4–BCAS3 and IRA1–RGS17*, as well as chimeric transcripts giving rise to fusion proteins (reviewed by Edwards, [142]).

### 4.10. Cancer Stem Cells in NSCLC and Breast Cancer

Cancer stem cells (CSC) have become an area of interest in a number of tumor types and are thought to be chemoresistant cells [143,144]. A commonly used marker of stem cells is CD133 (prominin-1) [145]. Using immunohistochemistry in primary lung cancers, an average percentage of epithelial cells expressing CD133 was found to be 5%, with a range of 0.02 to 35% CD133+ cells in lung cancer, as detected by flow cytometry [146]. Such cells are cisplatin resistant both *in vitro* and *in vivo*. Patients with CD133+ cells in their lung tumors tended to have a shorter progression-free survival [146]. Chemotherapeutic agents used commonly in NSCLC therapy result in enrichment of CSC [147], which are highly tumorigenic and metastatic [148]. Although CD133 is a marker of chemoresistance, it does not function as a prognostic marker for survival in NSCLC patients [149]. 

Breast CSCs have been identified by using a model in which human breast cancer cells were grown in immunocompromised mice. Only a minority of breast cancer cells with the phenotype CD44+ CD24-/low, starting with as few as 100 cells, were able to form tumors in NOD/SCID mice, whereas thousands of cells without this phenotype could not give rise to tumors in NOD/SCID mice [150]. High grade breast cancers have a higher content of CSCs than do low grade cancers [151].

### 4.11. Plasticity of Cancer Stem Cells

As each nucleated cell contains the entire genome of each individual, the maintenance of cellular normality, homeostasis, repair, and the maintenance or regeneration of organ structure, must be a complex and active process, and not an irreversible "turning off or on" of specific subsets of genes and their encoded proteins. In human cancers, the evidence for this includes metaplastic cancers [152], the inappropriate co-expression of lineage specific markers [153], and the maturation of cell types in certain paediatric sarcomas over time [154]. Cancer stem cells are thought to be pluripotent and thus could, over time, lead to outgrowth of cells with phenotypes that do not resemble the original clone. This fundamentally creates flaws in any type of classification system that is based on tissue of origin, cell type, phenotype, grade, *etc.*, as these assume that a pre-treatment snap-shot based upon static classification systems is capable of providing prognostic or predictive information about an individual patient with cancer. Complicating this is the fact that gene expression is controlled by the interaction of cells with their microenvironment, and *vice versa* [155,156,157,158,159,160,161]. Thus, instead of classifying cancers on the basis of organ of origin, cell type, differentiation, phenotype, *etc.*, we need to develop a functional, dynamic, pathway-based classification system that is agnostic of organ type or cellular origin, but more predictive of the probability of response to targeted therapies. Such classification systems would allow the selection of appropriate targeted therapies that are individualized and modified, as the tumor clones continue to evolve during therapy. 

### 4.12. Next Generation Sequencing Technology and Cancer Genomes

The functional classification of human cancers using current techniques is too cumbersome and expensive. The methods are numerous, each with a limited set of data available for developing a comprehensive map of pathway pathology in each individual cancer sample. Next generation sequencing, however, promises to significantly change this [162,163,164,165,166,167,168,169,170,171,172,173,174,175,176,177]. In the next few years, the cost and turnaround time of whole genome sequencing is expected to be competitive with conventional assays that provide only limited clinically useful information [166]. The technology has already allowed great insight into cancer cells and has enabled the recognition of novel markers of diagnostic importance [167,168,169,170,171,172,173,174,175,176,177,178,179,180]. If future comprehensive pathway analysis costs come down to around $1000 per patient as expected using the next generation sequencing technologies, the avoidable costs of ineffective therapies could reach up to CAN$1.1 billion per year for Canadian cancer patients (Table 3). Thus over a billion dollars (Canadian funds) could be redirected annually towards newer targeted therapies, which should yield better outcomes than the current "one size fits all" approach.

**Table 3:** Future ROI after test optimization example, in Canadian dollars.

	Patients to be tested per year	Test cost per year	ROI per year (Cost avoidance)	Net savings
Top 5 cancers	59,000	$59,000,000	$989,470,619	$930,470,619
Top 10 cancers	107,350	$107,350,000	$1,178,328,829	$1,070,978,829
Top 14 cancers	120,150	$120,150,000	$1,208,911,725	$1,088,761,725

Optimized assay cost per test (labor and materials), $1,000; ROI = Return on investment.

## 5. Conclusions

Intratumoral and intertumoral heterogeneity, tumor microenvironments, and individual genomes are likely to account for the variability of response to current therapies, conventional or targeted. Morphologic classification systems based upon the light microscopic features of human cancers are unlikely to be refined further, even with ancillary methods, such as immunohistochemistry and *in situ* hybridization, as the information gleaned from such methods will remain relatively limited in scope and prone to interobserver variability. In order to develop functional classification systems, we have to wean ourselves from static morphology based classification systems and adopt systems based upon pathway pathology maps generated by robust, massively parallel next generation sequencing technologies. Once such technologies become cost-effective and timely, they may consistently generate clinically reliable data that will allow personalized therapies. Due to the massive data sets that would be generated, advances in bioinformatics and computing power will be required. Bioinformaticians will need to be incorporated into the staffing plans for clinical laboratories for this to become the future of cancer diagnostics.
